# Seed Germination Response and Tolerance to Different Abiotic Stresses of Four *Salsola* Species Growing in an Arid Environment

**DOI:** 10.3389/fpls.2022.892667

**Published:** 2022-05-19

**Authors:** Pengyou Chen, Li Jiang, Weikang Yang, Lei Wang, Zhibin Wen

**Affiliations:** ^1^State Key Laboratory of Desert and Oasis Ecology, Xinjiang Institute of Ecology and Geography, Chinese Academy of Sciences, Urumqi, China; ^2^Sino-Tajikistan Joint Laboratory for Conservation and Utilization of Biological Resources, Urumqi, China; ^3^The Specimen Museum of Xinjiang Institute of Ecology and Geography, Chinese Academy of Sciences, Urumqi, China; ^4^University of Chinese Academy of Sciences, Beijing, China; ^5^Xinjiang Key Lab of Conservation and Utilization of Plant Gene Resources, Urumqi, China

**Keywords:** light, salinity, *Salsola*, seed germination, winged perianth, land rehabilitation

## Abstract

Land degradation caused by soil salinization and wind erosion is the major obstruction to sustainable agriculture in the arid region. *Salsola* species have the potential to prevent land degradation. However, there is limited information about seed germination requirements and tolerance to salinity and drought for representative *Salsola* species. This study aimed to assess the effects of the winged perianth (seed structural features) and abiotic factors (light, temperature, salinity, and drought) on the seed germination of these species. These *Salsola* species varied considerably in seed germination characteristics. Compared with naked seeds, winged seeds had lower germination percentages for *S. heptapotamica S. rosacea*, and *S. nitraria* species. Darkness decreased the germination percentage of winged and naked seeds of *S. rosacea*, however, for *S. heptapotamica* and *S. nitraria*, decreased seed germination was only when the winged perianth existed. Germination of *S. heptapotamica, S. rosacea*, and *S. nitraria* seeds depended on the perianth and light conditions. The naked seeds of these three species could germinate at a wide range of temperatures, especially in light. The presence of perianth, light, and temperature did not significantly influence the germination of *S. ruthenica* seeds. When cultivating these species, it is beneficial to remove the winged perianth of seeds and sow it on the soil surface when the temperature is above 5/15°C. In addition, seed germination of *Salsola* displayed high tolerance to salinity and drought. Compared with winged seeds, naked seeds showed lower recovery germination under high salinity but had a similar recovery of germination under high PEG concentration. Our study provides detailed germination information for the cultivation of these four representative *Salsola* species in degraded saline soils of the arid zone.

## Introduction

Land degradation refers to the succession process in which unfavorable natural factors or inappropriate land use leads to the gradual loss of production potential (Prince et al., 2009). The arid land in Central Asia is the largest dry area located in the temperate and warm temperate zone of the northern hemispheric earth, where land degradation is quite serious because of climatic variations and human activities resulting from population increase (Kuang et al., 2014). It is reported that the land degradation area in Central Asia is about 58.78 × 10^4^ km^2^, accounting for 10.37% of the total land area (Kuang et al., 2014). Salinity and drought are two abiotic factors resulting in land degradation in the arid region in Central Asia, which increasingly threaten sustainable agriculture with global climate change (Negrão et al., 2017). Currently, ~10% of the total land is degraded by salinity (Ruan et al., 2010) and up to 41% by drought (D'Odorico et al., 2013). Therefore, it is not unexpected that a large effort is devoted to repairing or restoring the deteriorating land (Zhang et al., 2015; Wang et al., 2020).

Cultivation of native plant species could be a viable alternative to rehabilitating such degraded land (El-Keblawy and Ksiksi, 2005). The use of suitable candidate plants is a vital step in any recovery program (El-Keblawy, 2017). One of the most important selection criteria to choose plants for land rehabilitation is their capacity of seeds to germinate under complex conditions, especially under stress (Lu et al., 2016). Therefore, a thorough knowledge of seed germination requirements for light and temperature, and tolerance to salinity or drought stress of native plants is the key aspect to understanding the role of the plants in the restoration and rehabilitation strategies (Qian et al., 2016).

Temperature is a determining factor for seed germination of most plants, which can break seed dormancy and stimulate germination (Probert, 1992; Baskin and Baskin, 2014). Seed germination of native plants in arid regions is often limited by the temperature though the other conditions are suitable (Evans and Etherington, 1990). Light is another important regulatory environmental signal for seed germination of desert plants (Katerina et al., 2014). In the desert and semi-desert areas, plants vary in their requirement for light during germination. Some germinate strictly in need of light (Sen and Chatterji, 1968), while others can germinate well in light or dark (Huang et al., 2003), and some even germinate better in dark than in light (Sekmen et al., 2004). In addition, the actual germination requirements of seeds to light depend on the interaction with other environmental factors such as temperature (El-Keblawy et al., 2011). Many desert plants germinate only when the combinations of light and temperature are suitable for seedling establishment (Naidoo and Naicker, 1992).

In arid and semi-arid regions, reduction in water potential of soil caused by salinity or drought is common stress that affects seed imbibition and thereby germination (Rasheed et al., 2019). Seed germination percentage is generally decreased with the increase in salinity or PEG concentration (Khan and Gulzar, 2003; Xing et al., 2013). Most ungerminated seeds remain viable under harsh conditions (e.g., high salinity and drought) by entering a state of conditional or enforced dormancy and can recover germination on the provision of sufficient moisture (Rasheed et al., 2015). Such germination inhibition may be a survival strategy for plants in arid regions, which could reduce seedling mortality (Khan and Gul, 2006).

There are a large group of plants in nature, whose seed structure has additional appendages such as winged perianth or bracteole (Jurado et al., 1991). Seeds with winged perianth take the advantage in dispersal (Baskin et al., 2014). Moreover, the presence of perianth-inhibited seed germination is seen in many plants, such as *Haloxylon stocksii* (Rasheed et al., 2019), *Salsola ikonnikovii* (Xing et al., 2013), and *S. schweinfurthii* (Bhatt et al., 2016). In general, inhibition of perianth on seeds germination could occur through many different pathways, such as induction of a light requirement for germination, mechanical inhibition, chemical inhibitors, and specific ion effects (Wei et al., 2008). In addition, the presence of perianth affected the germination response of seeds to different environmental factors, such as increased germination requirements to light of *S. rubescens* seed (El-Keblawy et al., 2013); change optimal temperature ranges of *H. stocksii* seed (Rasheed et al., 2019); and aggravate the inhibitive effect of salinity on seed germination of *S. ikonnikovii* (Xing et al., 2013), and of drought on seed germination of *S. ferganica* (Maimaitijiang et al., 2019).

*Salsola* L. belongs to the family Amaranthaceae and includes ~130 species occurring in the arid desert of Africa, Asia, and Europe (Zhu et al., 2003). There are ~37 species in China, 33 species of which distribute in Xinjiang. The typical feature of this genus is that perianth segments are abaxially winged in fruit (Zhu et al., 2003). Many plants of this genus have good effects on soil and water conservation, diminish wind- and sand-shifting (Wang et al., 2008), and thus have great potential for restoration of deteriorated land in arid regions. Some scholars have studied germination response to different environmental factors of seeds for *Salsola* plants (e.g., El-Keblawy et al., 2013; Elnaggar et al., 2019). The effects of winged perianth on seed germination are also considered (Wei et al., 2008; Ma et al., 2017). However, most of the researches only consider the effects of one or at most two factors between perianth, temperature and light on seed germination (Chang et al., 2008; Wang et al., 2013), comprehensive evaluation of the effects of these three factors are absent, which is vital to assess the true germination condition of seed in the arid desert. In addition, studies on the effect of perianth on seed germination tolerance to salinity or drought of *Salsola* plants are also limited (Xing et al., 2013; Maimaitijiang et al., 2019).

In this study, we collected seeds of four representative *Salsola* species, including three endemic species *S. heptapotamica Iljin, S. rosacea Linn*., and *S. nitraria Pall*, mainly distributed in northern Xinjiang in China, and one widespread species *S. ruthenica Iljin*. distributed in many places in China (Zhu et al., 2003). We performed laboratory germination tests and aimed to provide detailed germination information for better screening of suitable *Salsola* plants in rehabilitating deteriorated lands in arid lands in Central Asia. We hypothesize that (1) the presence of winged perianth inhibits seed germination of these species; (2) the germination responses to light and temperature of endemic species are more sensitive than that of widespread species; (3) all these *Salsola* species have a high tolerance to salinity and drought.

## Materials and Methods

### Seed Collection and Habitat Characteristics

Freshly matured fruits of the four *Salsola* species were collected from natural populations growing at the edge of Junggar Basin in Xinjiang during September–October 2020. This area is arid to semi-arid with a typical temperate continental climate. Annual precipitation is around 167 mm with minimum precipitation in winter and maximum in summer. Annual potential evaporation is 2,300 mm. The mean annual temperature is 6.7°C with a minimum temperature of −34.4°C in January and a maximum temperature 41.7°C in August. The soil is saline soil and the pH is above 8. Fruits of each species were randomly collected from about 50 plants and taken to the laboratory. Fruits were air-dried under room temperature (18–25°C) for 2 weeks and then were stored at 4°C for about 1 month until used in this experiment. The specific habitat characteristics are shown in Table 1.

**Table 1:** The habitat characteristics of four representative annual *Salsola* plants.

**Species**	**Habitat**	**Accompanying plants**	**Latitude and longitude**	**Altitude /m**
*S. heptapotamica* Iljin	Gobi desert, and sandy land	*S. affinis* C.A. Mey., *Haloxylon ammodendron* (C. A. Mey.) Bunge, *Kalidium foliatum* (Pall.) Moq.	N44°45'E87°53'	370
*S. rosacea* Linn.	Gravelly soil before the mountain	*S. collina* Pall., *Anabasis salsa* (C. A. Mey.) Benth. ex Volkens, *Atriplex*	N43°44'E82°55'	810
*S. nitraria* Pall	Gravelly soil	*H. ammodendron* (C. A. Mey.) Bunge, *Halogeton glomeratus* (Bieb.) C. A. Mey.	N44°48'E86°37'	320
*S. ruthenica* Iljin	Sandy soil of valley and gravel gobi	*Artemisia, S. affinis* C.A. Mey., *A. micrantha* C. A. Mey.	N44°19'E87°57'	400

### Seed Characteristics

The size, shape, and color of the four *Salsola* seeds with and without winged perianth (hereafter as winged seeds and naked seeds) were recorded using the stereomicroscope (Olympus SZX10, Tokyo, Japan). Winged perianth-enclosed seeds were removed manually. Seven groups of 100 winged seeds or naked seeds were weighed using an analytical balance (precision 0.001 g). The sizes of winged seeds or naked seeds were determined using Image J Analysis Software (National Institutes of Mental Health, America). Each determination had seven replicates.

### Seed Germination

#### Winged Perianth, Light, and Temperature Effects

The winged seeds or naked seeds were incubated in five incubators adjusted to a temperature regime of 5/15, 5/20, 10/25, 15/30, and 20/35°C (common temperature regimes of the region) in both continuous darkness and alternating 12 h darkness/12 h light, hereafter referred as dark and light, respectively. Seeds were placed in 9-cm-diameter Petri dishes on two layers of Whatman no. 1 filter paper moistened with 7 ml of distilled water. The Petri dishes were sealed with parafilm. For the dark treatment, the Petri dishes were wrapped in two layers of aluminum foil to prevent any exposure to light. Each treatment had four replicates with 25 seeds. Radicle protrusion from the seed was the criterion for germination of winged seeds, and naked seeds were germinated when the radicle length was ≥ 2 mm. Seed germination was monitored every day, germinated seeds were discarded at each counting, and the experiment lasted for 14 d. The seeds incubated in dark were checked only after 14 d. The rate of germination was estimated by using a modified Timson's index of germination velocity = ΣG/t, where G is the percentage of seed germination every day and t is the total germination period (Khan and Ungar, 1984).

#### Winged Perianth and Salinity Effects

Due to the relatively higher germination percentages of the four *Salsola* species in light and at 10/25°C, winged seeds and naked seeds were sown in different NaCl concentrations (0, 50, 100, 300, 500, and 700 mM, based on preliminary results) and incubated at this condition. Each dish was wrapped with parafilm against loss of water by evaporation. Germination was monitored after 14 d of incubation. All the seeds that failed to germinate were rinsed with distilled water and then incubated in distilled water for another 7 d. Recovery percentages (RP) were calculated using the formula: RP = [(a – b)/c] × 100, where a is the sum of the number of seeds germinated in NaCl solutions plus those that recovered to germinate in the distilled water; b is the total number of seeds germinated in NaCl solutions, and c is the total number of seeds tested. The total germination percentage was recorded as (a/c) × 100.

#### Winged Perianth and Drought Effects

Winged seeds or naked seeds were germinated in Petri dishes with six levels of polyethylene glycol (PEG) concentrations (0, 50, 100, 150, 200, 300 g·L^−1^, based on preliminary results), which correspond to osmotic potential (Ψ_S_) of 0, −0.05, −0.15, −0.3, −0.5 and −1.03 MPa (Michel and Kaufmann, 1973). The light and temperature conditions were the same as in the salinity experiment. Recovery percentage and total germination were also calculated.

### Statistical Analysis

Seed germination data are of binomial type (i.e., germination is 1, not germination is 0). The forward stepwise (Wald) in binary logistic regression models was used to analyze the effect of winged perianth, light, temperature, and their interactions on seed germination. The same method was used to determine the effect of the winged perianth, salinity and their interactions, winged perianth, PEG and their interactions on seed germination and total germination. This method will automatically eliminate the parameters with a low probability of Wald statistics in the equation, to ensure the accuracy of the regression curve to a great extent (Li and Luo, 2003). In addition, for parameters that have significant effects on germination, Turkey's multiple comparison tests were used to test the differences that existed among groups. The same method was also used to determine the effect of winged perianth and temperature on the germination index of these *Salsola* species. Non-parametric tests were also performed to test the differences among the recovery percentages that did not meet the homogeneity of variance in different concentrations of NaCl or PEG solution. The difference between perianth treatments at the same temperature of seed germination was analyzed by Student's test at 95% confidence level. Data were expressed as mean ± standard error. All statistical tests were analyzed using the IBM SPSS Statistics 20 (IBM Corp., Armonk, New York, United States).

## Results

### Seed Characteristics

The winged seeds of the four *Salsola* plants are all utricle and surrounded by fan-shaped and membranous wings (Supplementary Figures 1B–K). The naked seeds of the four plants are slightly flattened to slightly conical and have the typical spiral embryo (Supplementary Figures 1C–L). Winged seeds and naked seeds of *S. heptapotamica* and *S. rosacea* are significantly larger and heavier than those of *S. nitraria* and *S. ruthenica* (Supplementary Figure 1; Table 2).

**Table 2:** The characteristics of winged seed and naked seed of the four annual *Salsola* plants.

**Species**	**Radius of winged seed (mm)**	**Diameter of naked seed (mm)**	**Mass/100 winged seeds (g)**	**Mass/100 naked seeds (g)**
*S. heptapotamica*	5.627 ± 0.154^a^	2.671 ± 0.088^a^	0.751 ± 0.009^a^	0.391 ± 0.005^a^
*S. rosacea*	4.894 ± 0.106^b^	2.387 ± 0.111^b^	0.514 ± 0.004^b^	0.307 ± 0.005^a^
*S. nitraria*	3.674 ± 0.151^c^	1.024 ± 0.037^c^	0.192 ± 0.002^c^	0.064 ± 0.001^b^
*S. ruthenica*	2.339 ± 0.214^d^	1.116 ± 0.025^c^	0.155 ± 0.002^c^	0.083 ± 0.001^b^

*Data are the mean of seven replicates (± SE). Different letters in columns indicate significant differences (p < 0.05) among the four Salsola plants*.

### Winged Perianth, Light, and Temperature Effects

**Figure 1:**
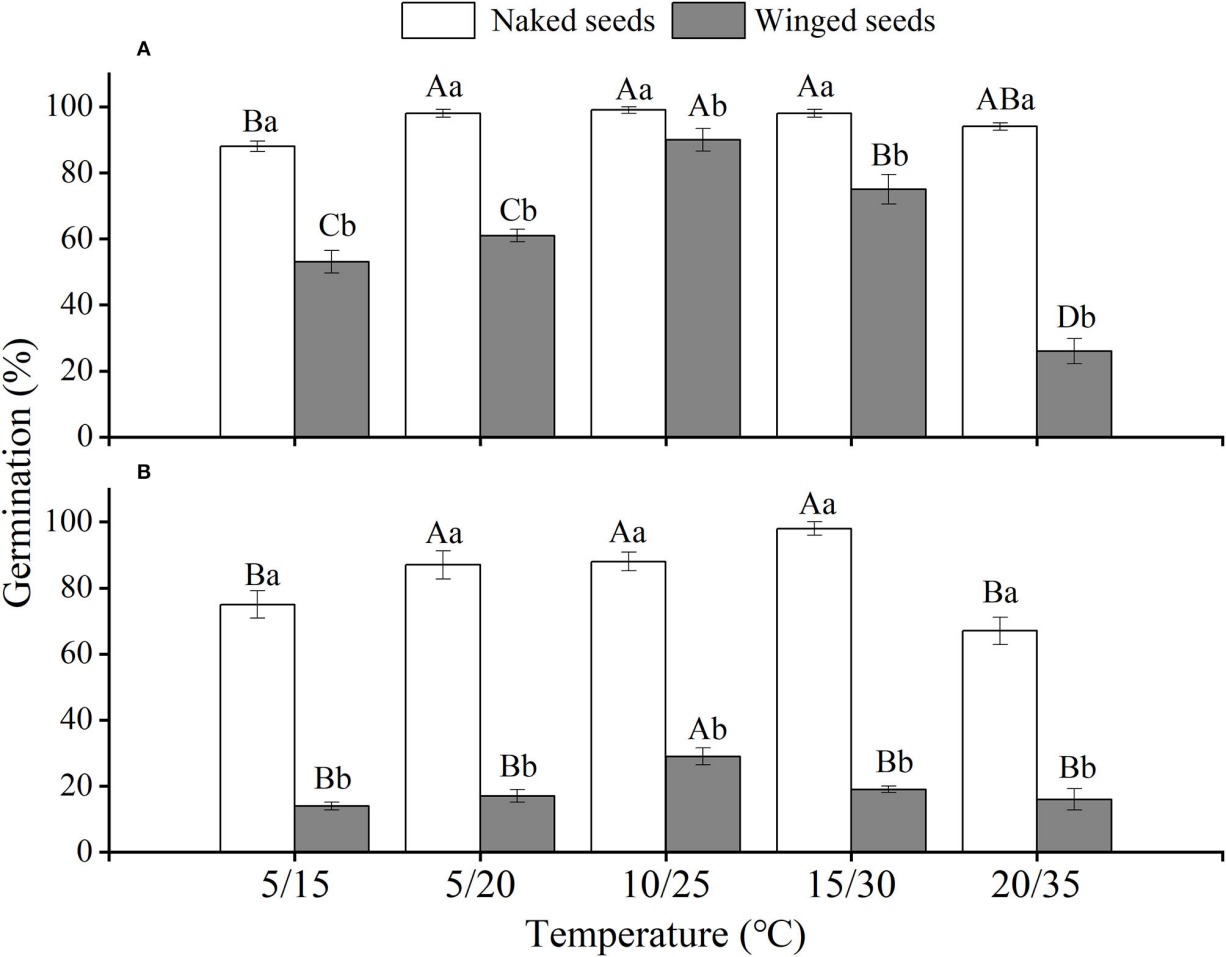
The effects of winged perianth, light, and temperature on seed germination percentage (mean ± SE) of *Salsola heptapotamica*. **(A)** germination in light, **(B)** germination in dark. Different uppercase letters denote a significant difference (*p* < 0.05) in germination percentage at different temperatures for the same perianth treatment, and different lowercase letters indicate a significant difference (*p* < 0.05) of germination percentage for different treatments of perianth at the same temperature.

**Figure 2:**
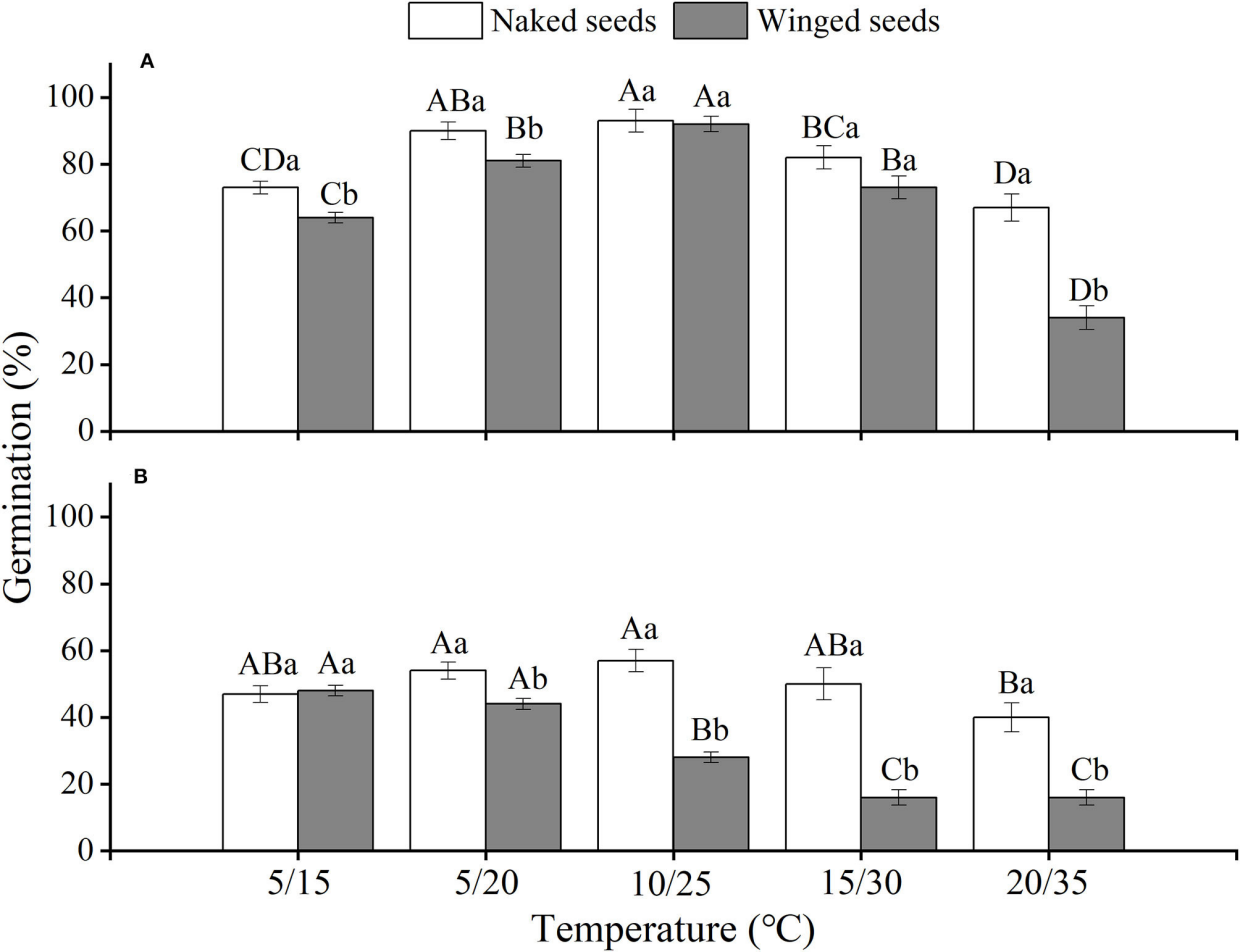
The effects of winged perianth, light, and temperature on seed germination percentage (mean ± SE) of *Salsola rosacea*. **(A)** germination in light, **(B)** germination in dark. Different uppercase letters denote a significant difference (*p* < 0.05) in germination percentage at different temperatures for the same perianth treatment, and different lowercase letters indicate a significant difference (*p* < 0.05) of germination percentage for different treatments of perianth at the same temperature.

**Figure 3:**
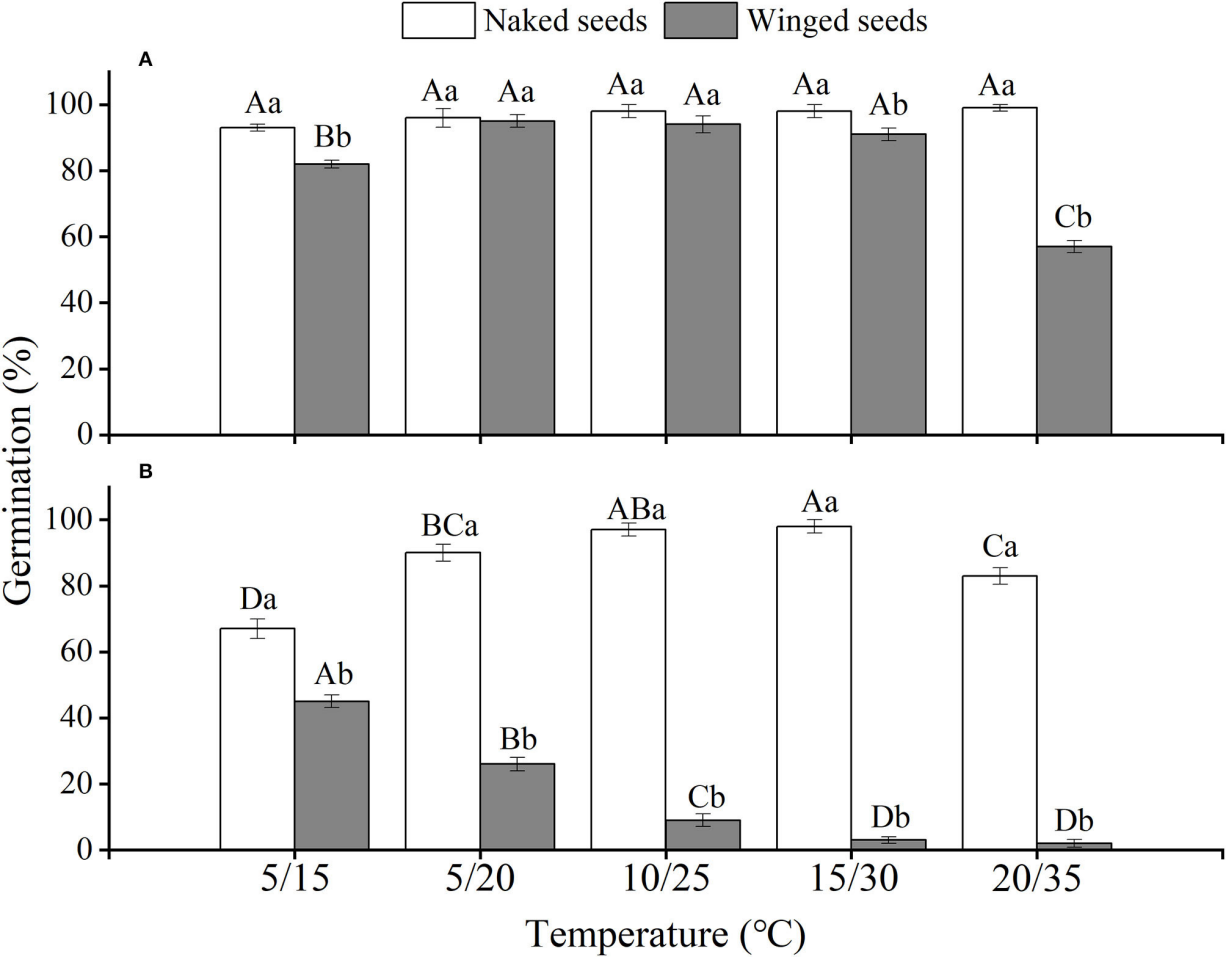
The effects of winged perianth, light, and temperature on seed germination percentage (mean ± SE) of *Salsola nitraria*. **(A)** germination in light, **(B)** germination in dark. Different uppercase letters denote a significant difference (*p* < 0.05) in germination percentage at different temperatures for the same perianth treatment, and different lowercase letters indicate a significant difference (*p* < 0.05) of germination percentage for different treatments of perianth at the same temperature.

**Figure 4:**
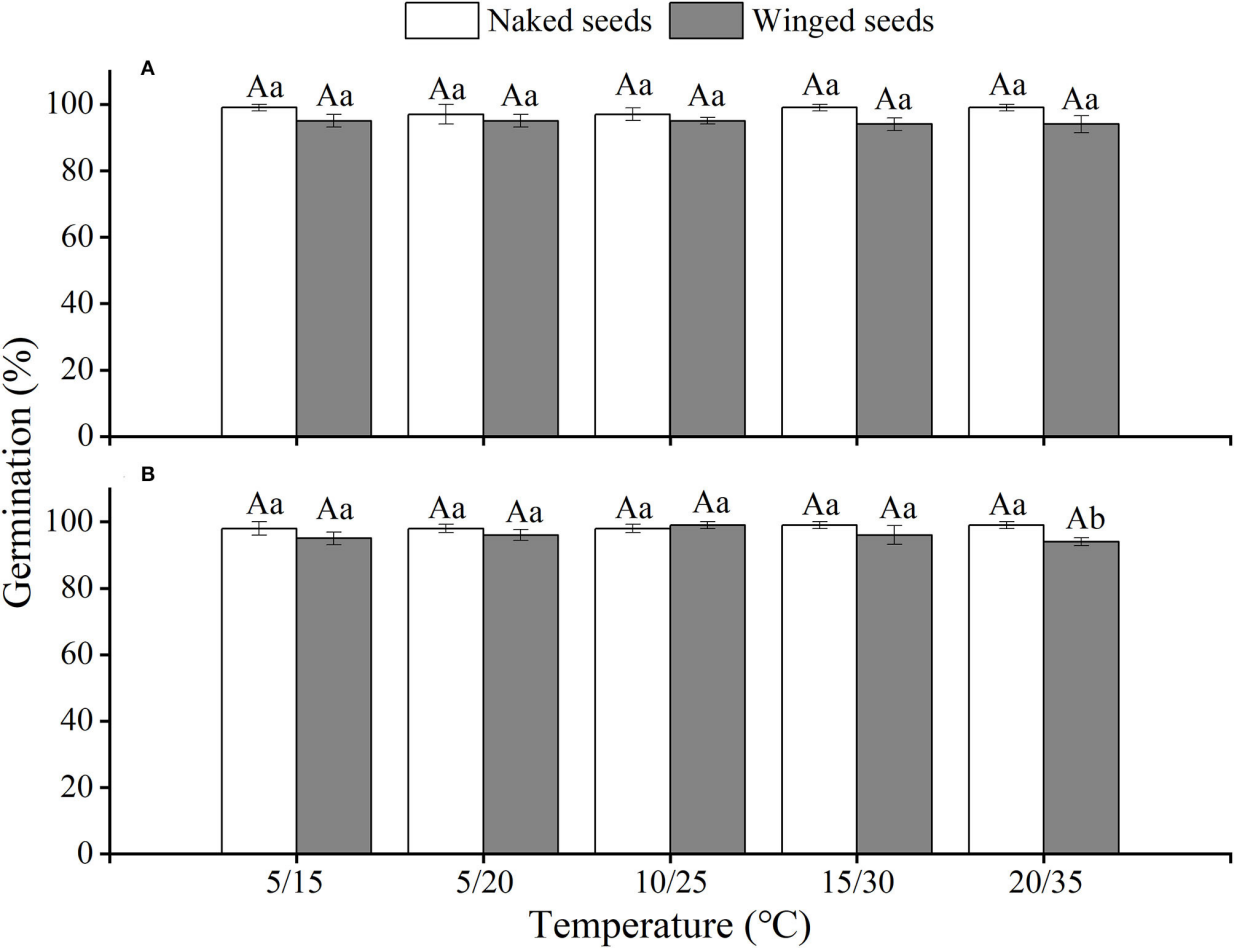
The effects of winged perianth, light, and temperature on seed germination percentage (mean ± SE) of *Salsola ruthenica*. **(A)** germination in light, **(B)** germination in dark. Different uppercase letters denote a significant difference (*p* < 0.05) of germination percentage at different temperatures for the same perianth treatment, and different lowercase letters indicate a significant difference (*p* < 0.05) of germination percentage for different treatments of perianth at the same temperature.

The effects of winged perianth, light, and temperature on seed germination of *S. heptapotamica, S. rosacea*, and *S. nitraria* were significant. The interactive effect of winged perianth, light, and temperature on seed germination of *S. heptapotamica* and *S. nitraria* were also significant but were not for *S. rosacea* (Supplementary Table 1). For the three *Salsola* plants, germinations of winged seeds were all significantly lower than those of naked seeds (Figures 1–3). Germination percentages of winged and naked seeds of *S. rosacea* were significantly lower in dark than those in the light (Figures 2A,B). While for *S. heptapotamica* and *S. nitraria*, only winged seeds performed significantly lower germination percentages in dark than in light (Figures 1A,B, 3A,B). At the five temperature ranges, germination percentages of naked seeds for the three *Salsola* plants were all up to 40% whether in light or dark. For winged seeds germinated in dark, germination percentages were all no more than 43%, and winged seeds of *S. nitraria* were even <5% at 15/30–20/35°C (Figures 1–3). For *S. ruthenica*, except winged perianth (*p* < 0.001), other factors and their interactions had no significant effect on seed germination (Supplementary Table 1). Besides, germination percentages of winged and naked seeds were both more than 90% at the five temperatures whether in light or dark (Figures 4A,B).

For *S. heptapotamica, S. rosacea*, and *S. nitraria*, germination indices of winged seeds were significantly lower than those of naked seeds (Supplementary Figures 2A–C). Germination indices of naked seeds for these three *Salsola* plants were increased with the raising of temperature, but those for winged seeds were increased initially and then decreased (Supplementary Figures 2A–C). For *S. ruthenica*, there was no significant difference in germination indices between winged and naked seeds at the five temperatures (Supplementary Figure 2D).

### Winged Perianth and Salinity Effects

**Figure 5:**
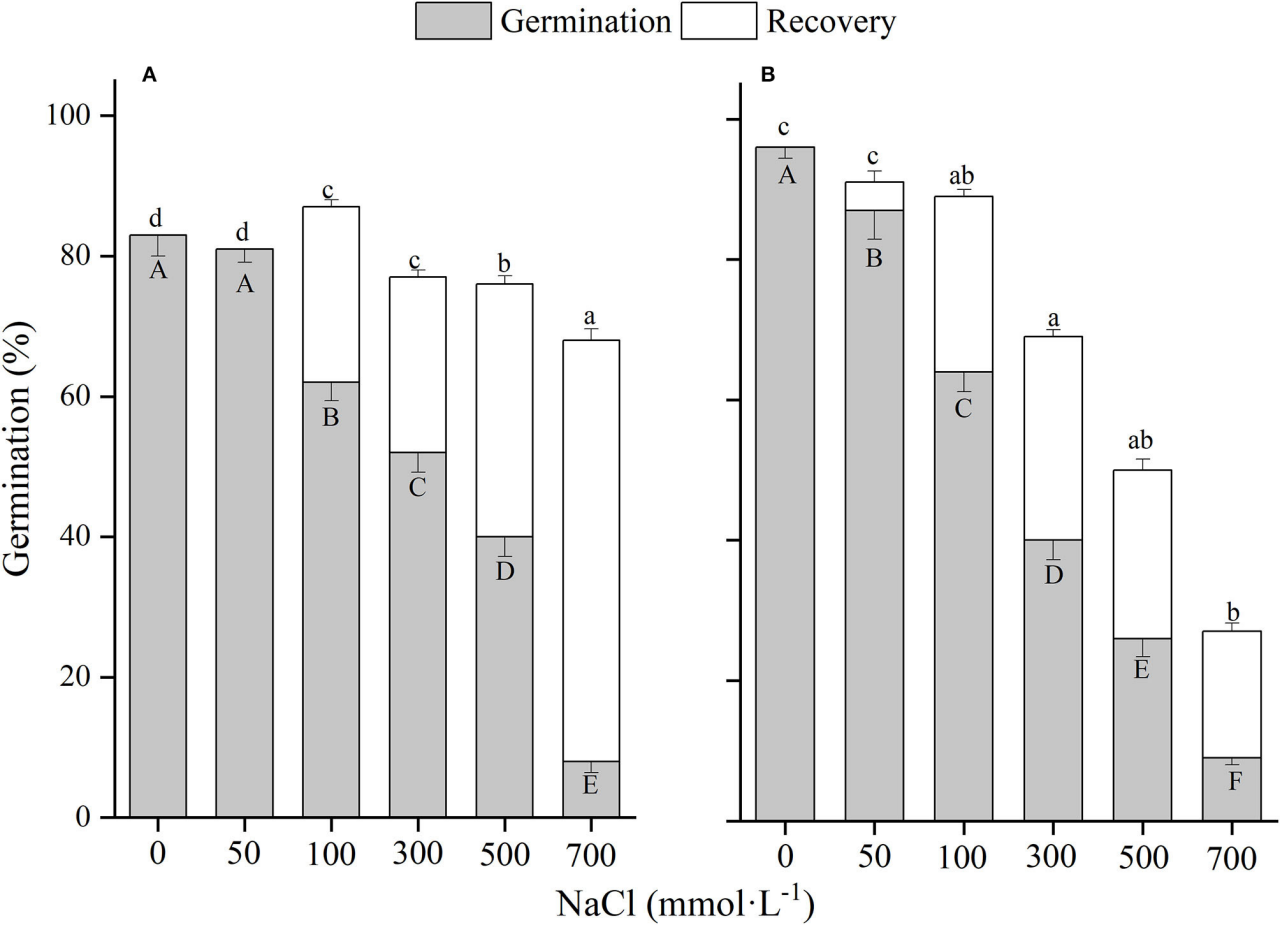
The effects of winged perianth and NaCl concentration on seed germination and recovery germination of *Salsola heptapotamica*. **(A)** winged seeds, **(B)** naked seeds. Different uppercase letters denote a significant difference (*p* < 0.05) in germination percentage among different NaCl concentration. Different lowercase letters indicate a significant difference (*p* < 0.05) in recovery germination among different NaCl concentration.

**Figure 6:**
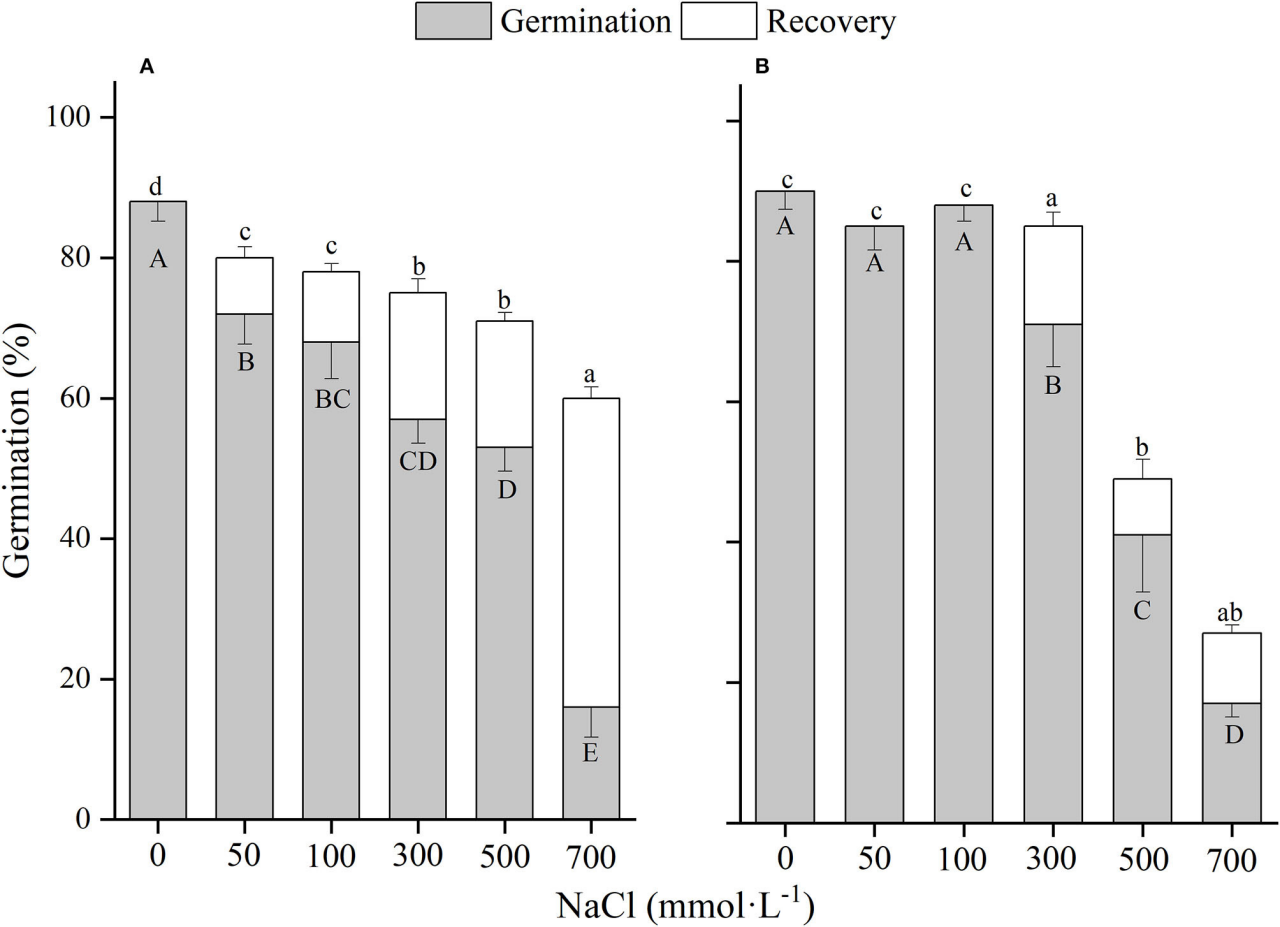
The effects of winged perianth and NaCl concentration on seed germination and recovery germination of *Salsola rosacea*. **(A)** winged seeds, **(B)** naked seeds. Different uppercase letters denote a significant difference (*p* < 0.05) in germination percentage among different NaCl concentrations. Different lowercase letters indicate a significant difference (*p* < 0.05) in recovery germination among different NaCl concentrations.

**Figure 7:**
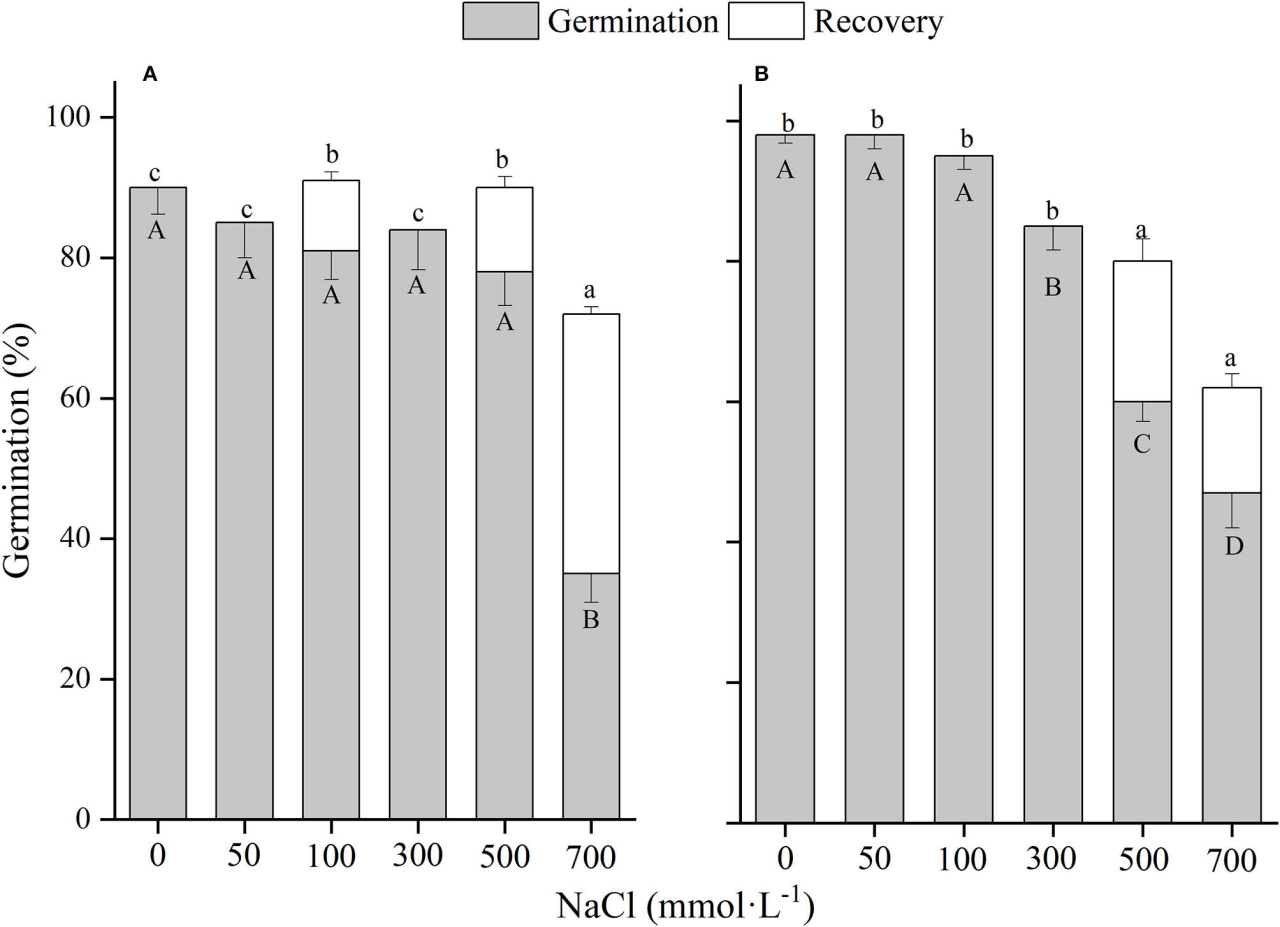
The effects of winged perianth and NaCl concentration on seed germination and recovery germination of *Salsola nitraria*. **(A)** winged seeds, **(B)** naked seeds. Different uppercase letters denote a significant difference (*p* < 0.05) in germination percentage among different NaCl concentrations. Different lowercase letters indicate a significant difference (*p* < 0.05) of recovery germination among different NaCl concentrations.

**Figure 8:**
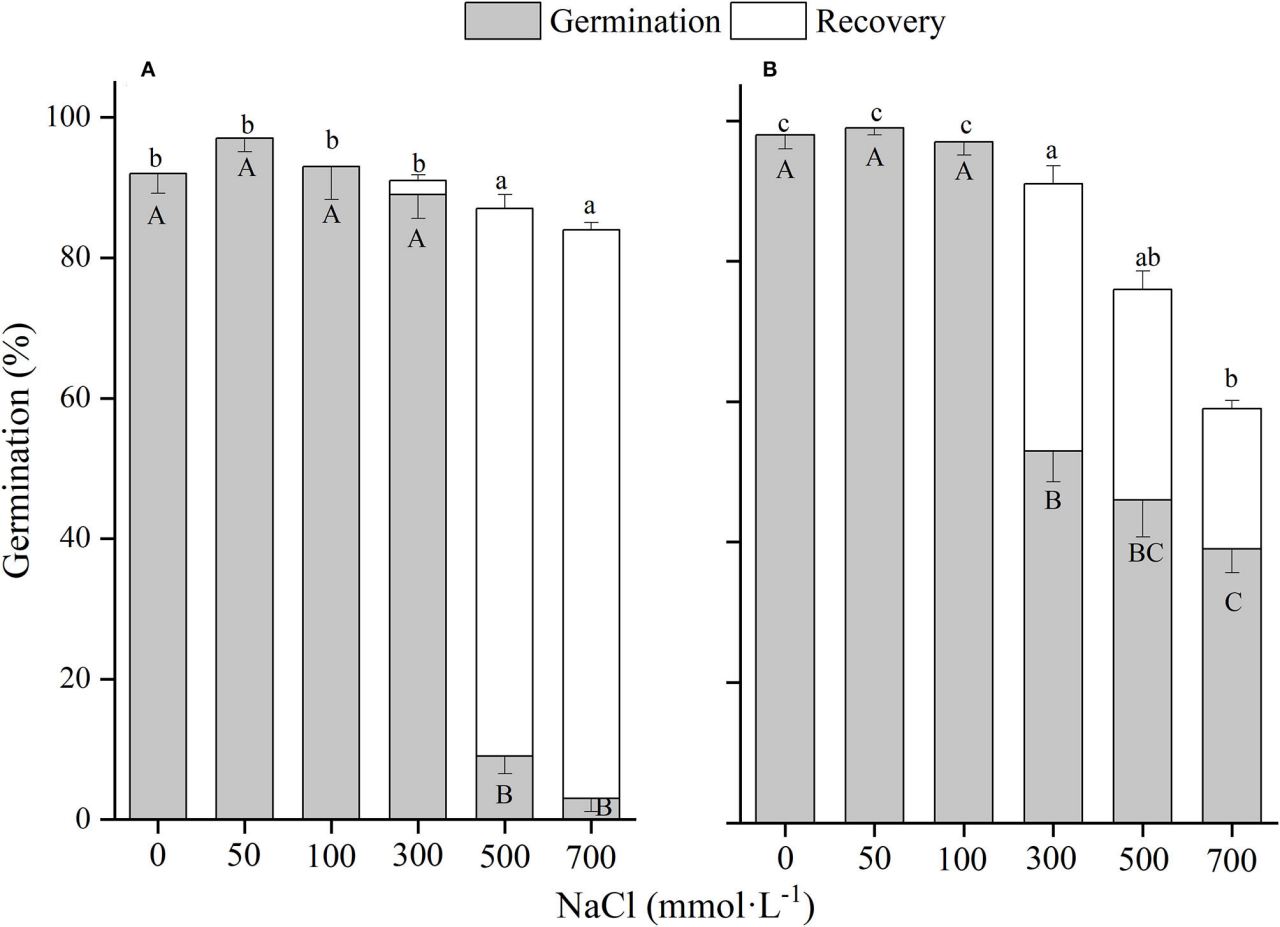
The effects of winged perianth and NaCl concentration on seed germination and recovery germination of *Salsola ruthenica*. **(A)** winged seeds, **(B)** naked seeds. Different uppercase letters denote a significant difference (*p* < 0.05) in germination percentage among different NaCl concentrations. Different lowercase letters indicate a significant difference (*p* < 0.05) of recovery germination among different NaCl concentrations.

There was a significant (*p* < 0.01) interaction of winged perianth and salinity on seed germination and total germination for the four *Salsola* plants (Supplementary Table 2). Germination percentages and total germinations of winged and naked seeds of the four *Salsola* plants were all decreased with the increase of NaCl concentration (Figures 5–8). Germination percentages of naked seeds were all higher than those of winged seeds, except at 500 mM, in which germination percentages of naked seeds for *S. heptapotamica, S. rosacea*, and *S. nitraria* were lower than those of winged seeds (Figures 5–7). The recovery percentages of winged seeds for the four *Salsola* were all increased with the raising of NaCl concentration, while those of naked seeds were increased initially and then decreased. At 0–300 mM NaCl concentration, the total germinations of naked seeds for the four *Salsola* plants were higher than those of winged seeds or there were no significant differences, but at 500–700 mM, the results were opposite (Figures 5–8).

### Winged Perianth and Drought Effects

**Figure 9:**
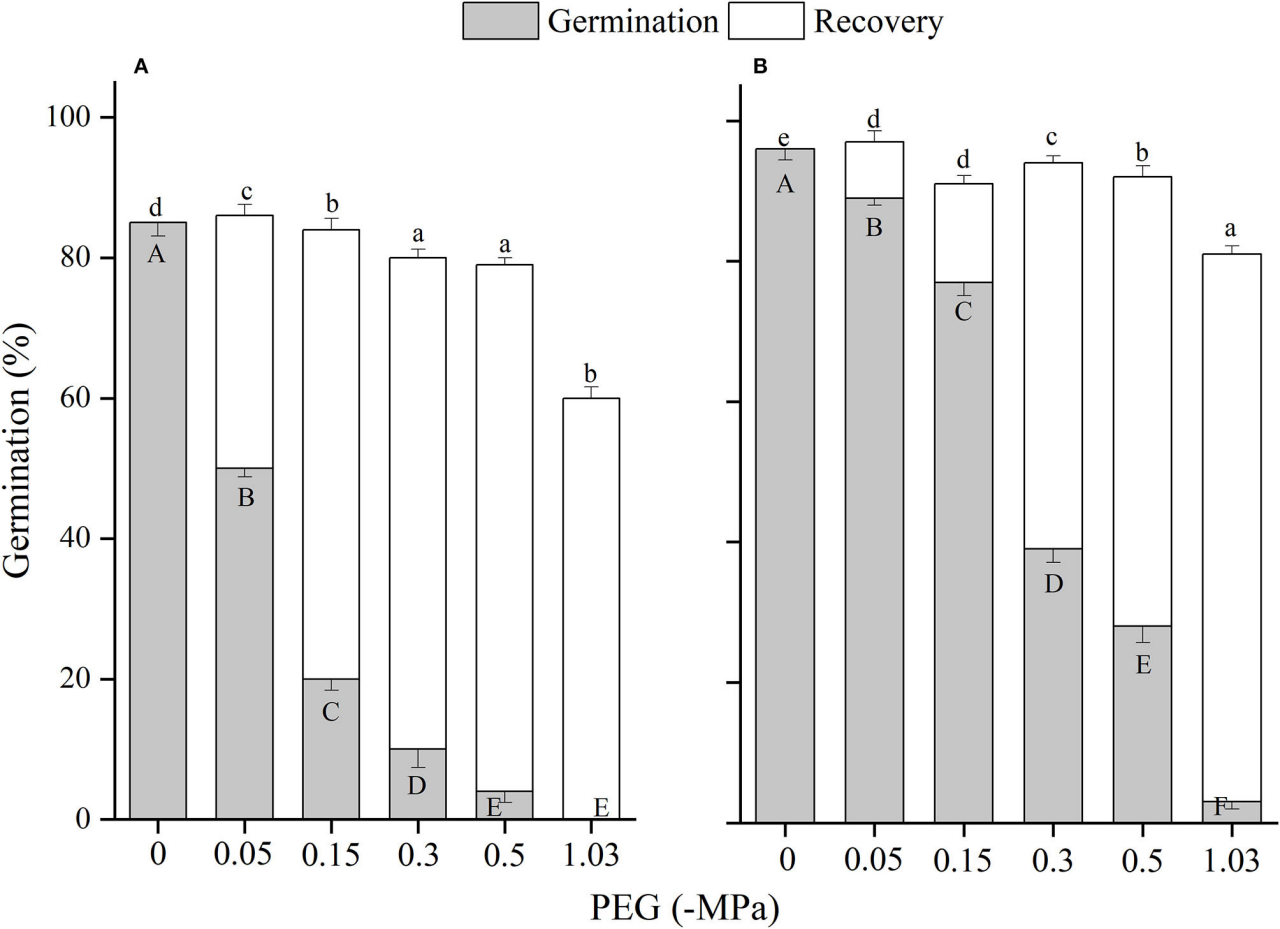
The effects of winged perianth and PEG concentration on seed germination and recovery germination of *Salsola heptapotamica*. **(A)** winged seeds, **(B)** naked seeds. Different uppercase letters denote a significant difference (*p* < 0.05) in germination percentage among different PEG concentrations. Different lowercase letters indicate a significant difference (*p* < 0.05) of recovery germination among different PEG concentrations.

**Figure 10:**
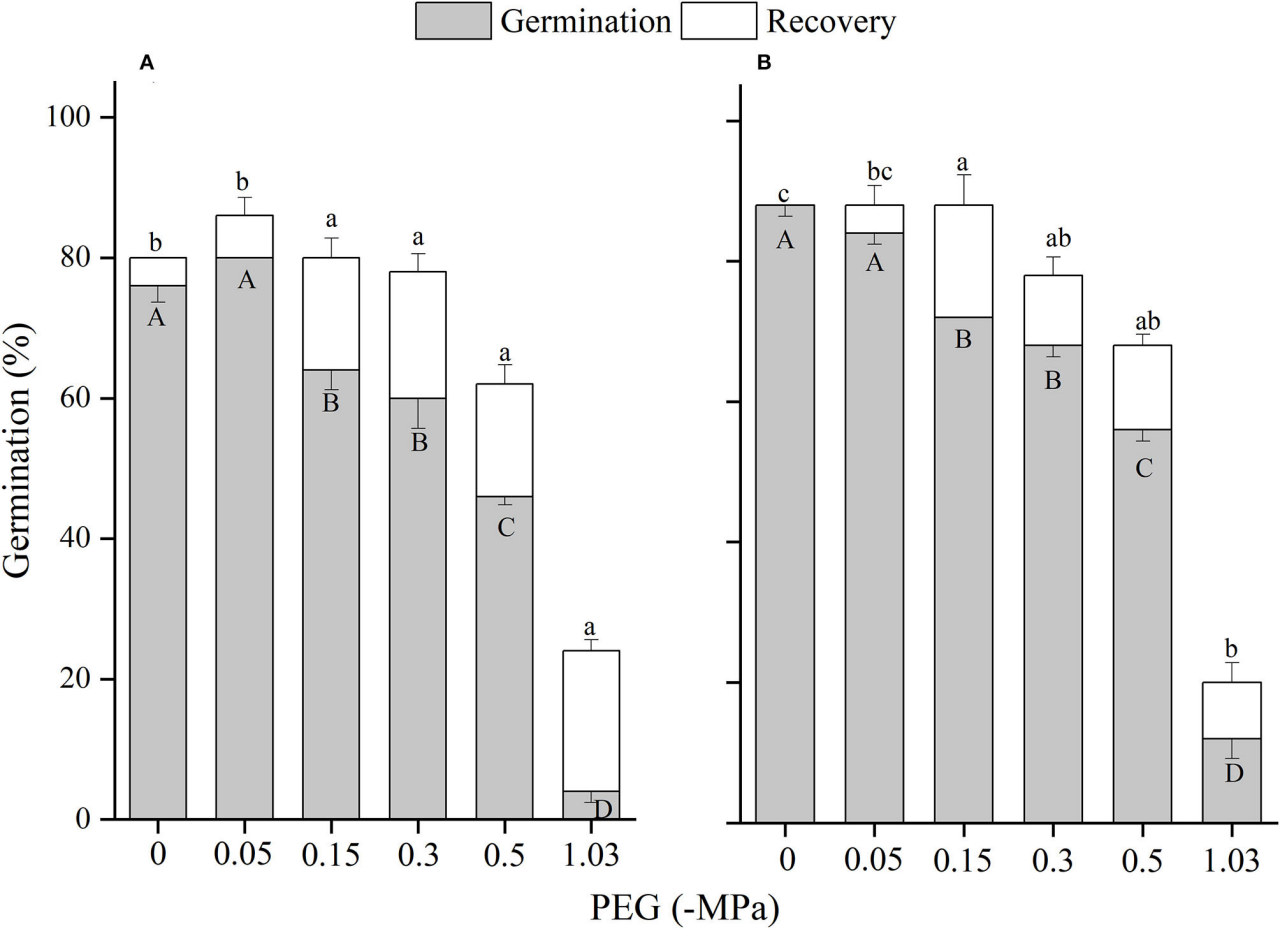
The effects of winged perianth and NaCl concentration on seed germination and recovery germination of *Salsola rosacea*. **(A)** winged seeds, **(B)** naked seeds. Different uppercase letters denote a significant difference (*p* < 0.05) in germination percentage among different PEG concentrations. Different lowercase letters indicate a significant difference (*p* < 0.05) in recovery germination among different PEG concentrations.

**Figure 11:**
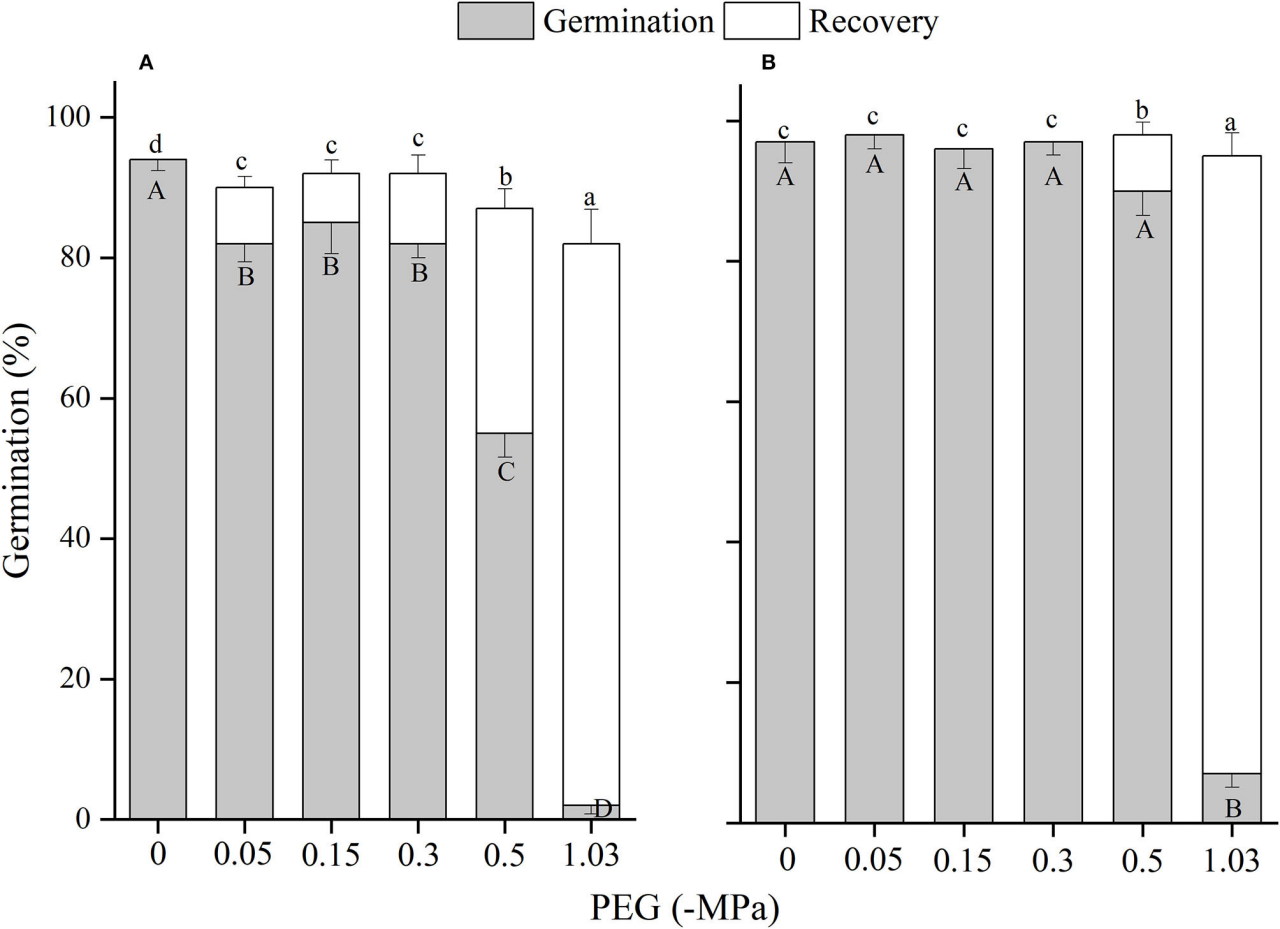
The effects of winged perianth and NaCl concentration on seed germination and recovery germination of *Salsola nitraria*. **(A)** winged seeds, **(B)** naked seeds. Different uppercase letters denote a significant difference (*p* < 0.05) in germination percentage among different PEG concentration. Different lowercase letters indicate a significant difference (*p* < 0.05) in recovery germination among different PEG concentrations.

**Figure 12:**
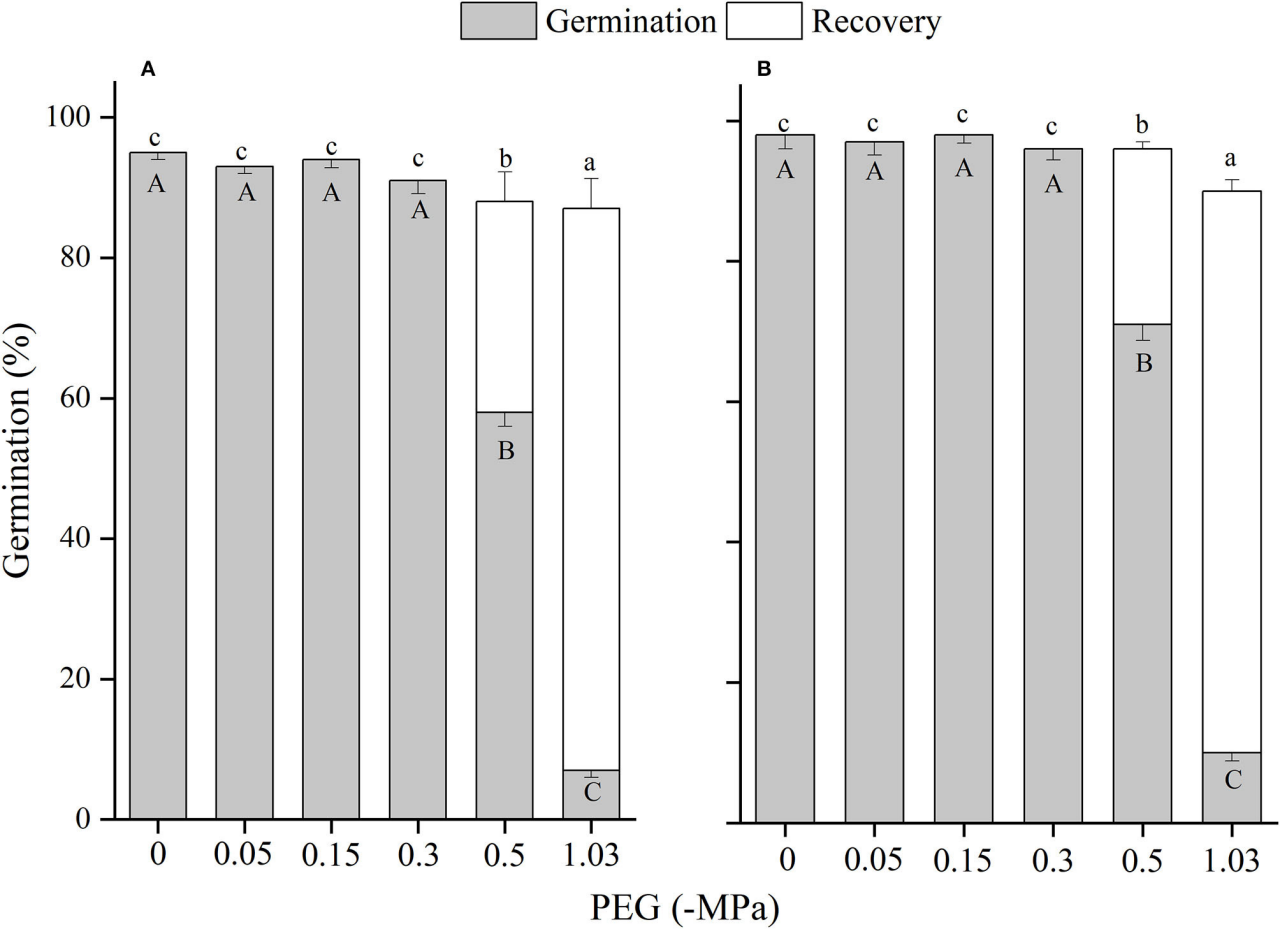
The effects of winged perianth and NaCl concentration on seed germination and recovery germination of *Salsola ruthenica*. **(A)** winged seeds, **(B)** naked seeds. Different uppercase letters denote a significant difference (*p* < 0.05) in germination percentage among different PEG concentrations. Different lowercase letters indicate a significant difference (*p* < 0.05) in recovery germination among different PEG concentrations.

There were significant (*p* < 0.05) effects of winged perianth and drought on seed germination of the four *Salsola* species, but were not for their interactive effects. There was no significant interaction of winged perianth and drought on total germinations of the four *Salsola* plants either (Supplementary Table 3). Germination percentages and total germination of winged and naked seeds for the four *Salsola* plants were all decreased with the decline in Ψ_S_ of PEG solution (Figures 9–12). Germination percentages and total germinations of naked seeds were all higher or there were no significant differences than those of winged seeds. The winged seeds of *S. heptapotamica* even had no germination at Ψ_S_ of −1.03 MPa (Figure 9A). The recovery percentages of winged and naked seeds for *S. heptapotamica, S. nitraria*, and *S. ruthenica* were all increased with the decrease of Ψ_S_, while those for *S. rosacea* seeds were increased initially and then decreased (Figure 10B).

## Discussion

Although there is some information about the germination characteristics of *Salsola* seeds, our data provide a thorough knowledge of the seed germination ecology of four representative *Salsola* species to complex abiotic conditions and the potential interactions with the winged perianth. These data also indicate that the presence of winged perianth inhibits seed germination of *S. heptapotamica, S. rosacea*, and *S. nitraria*. In addition, all these *Salsola* species have a high tolerance to salinity and drought. The results indicate that the germination responses to light and temperature conditions of endemic species are more sensitive than that of widespread species.

Perianth-enclosed seeds are a characteristic feature of the *Salsola* species, which helps in the dispersal of seeds by wind. But the presence of winged perianth usually inhibit seed germination (El-Keblawy et al., 2013). In this study, germination percentages and germination indices of winged seeds of *S. heptapotamica, S. rosacea*, and *S. nitraria* were significantly inhibited. Removal of winged perianth significantly increased the germination percentages and germination indices of the three *Salsola* plants. A similar effect of perianth on seeds germination was also found in *H. persicum* (Wei and Wang, 2006), *S. affinis* (Wei et al., 2008), and *H. stocksii* (Rasheed et al., 2019). Previous studies reported that perianth-inhibited seed germination by acting as a mechanical barrier for radicle emergence or due to the presence of inhibitor substances, which caused low germinability of seeds (El-Keblawy et al., 2013; Xing et al., 2013). In addition, our results showed that inhibitory effects of winged perianth on seed germination could be alleviated at some specific combination of temperature and light, such as germinating at 10/25°C and light, indicating winged perianth has complex effects on germination requirements to light and temperature.

For *S. rosacea*, germination percentages of winged and naked seeds were all significantly lower in dark than in light at the five temperatures, which indicated the positive photoblastic nature of the seeds. Light requirement of seed ensures germination at or near soil surface that facilitates seedling survival and growth (Rasheed et al., 2019). For *S. heptapotamica* and *S. nitraria*, compared with naked seeds, only germinations of winged seeds showed high light requirement, indicating that presence of winged perianth enhanced seeds germination requirements to light for these two *Salsola* plants. Light-filtering properties of the winged perianth surrounding the seeds might be responsible for the sensitivity of germination to light (Cresswell and Grime, 1981). The color of the winged perianth of *S. nitraria* was yellow-green. The relative lower light absorbance by winged perianth of *S. nitraria* might be the reason for greater light requirement during seeds germination. However, the winged perianth of *S. heptapotamica* has a variety of colors in the wild (Zhu et al., 2003), and the absorption of pigments to light is not enough to explain the high light requirement for seed germination, which is needed to further research.

The naked seeds of *S. heptapotamica, S. rosacea*, and *S. nitraria* had up to 60% germination percentages in light, and had more than 40% germination percentages in dark at all tested temperature regimes. While winged seeds for these three *Salsola* plants were hard to germinate in dark, with <5% germination percentage for *S. nitraria* at high temperatures. These results indicated significant interaction among winged perianth, light, and temperature for seeds germination. In many species, interactions among different factors are often more inhibitory for seed germination than their individual effects (Rasheed et al., 2019). In this study, the inhibitory effect of winged perianth on seed germination aggravated further in dark at unsuitable temperatures.

Germination percentage of winged and naked seeds for *S. ruthenica* was almost 100% at wide temperature regimes from 5/15 to 20/35°C whether in light or dark. This indicated that *S. ruthenica* seeds may have an “opportunistic” germination strategy that allowed them to highly germinate in relatively wide environmental conditions (Wei et al., 2007), which might be the reason for its wide distribution in the Central Asia region (Zhu et al., 2003). In a changing climate, a wide adaptation range of environments confers an advantage for the species to be used in rehabilitation. Besides, germination indices of *S. ruthenica* seeds were more than 90% at the five temperatures, which allowed them to rapidly develop into seedlings in early spring when the soil is sufficiently moist, to occupy favorable habitats and complete colonization in arid deserts. By contrast, germination percentages and germination indices of *S. heptapotamica, S. rosacea*, and *S. nitraria* seeds were relatively low due to their interaction of winged perianth, light, and temperatures, which prevented germination of all the seeds at one time. Retention of a fraction of ungerminated seeds in the seed bank could be considered a bet-hedging strategy to share the risk of seed germination and improve chances for survival (Saatkamp et al., 2011). However, it also prevents these three species from being widely distributed as *S. ruthenica* in different habitats (Zhu et al., 2003).

These four *Salsola* plants decreased germination percentages with the increase of NaCl concentrations. Similar results are also found in other species, such as *Suaeda salsa* (Song et al., 2008) and *S. iberica* (Khan et al., 2002). This effect might be caused by salinity-induced osmotic stress and/or ion toxicity (Song et al., 2005). Seeds of the four *Salsola* plants can germinate at 700 mM NaCl solution with >10% germination percentages, which showed higher tolerance to salinity than other halophytes, such as *S. vermiculata* 600 mM (Guma et al., 2010), *Atriplex triangularis* 510 mM (Khan and Ungar, 1984), and *Halogenton glomeratus* 400 mM (Ahmed and Khan, 2010). Besides, El-Keblawy et al. (2020) found that seeds germination of *S. drummondii* collected in non-salty habitats had a lower germination percentage than those in salty habitats, indicating that maternal environment played an important role in seed tolerance to salinity. In our study, seeds of the four *Salsola* plants were all collected from non-salty habitats, which may be underestimated of their salt tolerance. In addition, the presence of winged perianth aggravated the inhibition effect of NaCl on germination percentage, especially for *S. ruthenica*, of which germination of naked seeds (39%) was significantly higher than that of winged seeds (3%) at 700 mM NaCl concentration. However, the recovery germinations and total germinations of winged seeds for the four *Salsola* plants at higher NaCl concentrations were significantly higher than those of naked seeds, suggesting that winged perianth also plays a positive role in the protection of seeds during exposure to high salinity (Xing et al., 2013).

Drought stress also caused a reduction in seed germination percentage and total germination of the four *Salsola* plants, which might be ascribed to a reduction in the osmotic potential of the solution that restricts sufficient imbibition of seeds (Tobe et al., 2000). At Ψ_S_ of −1.03 MPa, except for *S. heptapotamica* with the least germination percentage of 3%, the other three *Salsola* plants can germinate ~10%, which were more tolerant to drought than *Agropyron mongolicum* and *Caragana korshinskii* (Yu et al., 2021), *S. vermiculata* (Al-Shamsi et al., 2018), and *Lachnoloma lehmannii* (Mamut et al., 2019). Moreover, the inhibitive effect of drought was performed more significantly by the presence of winged perianth. The germination of winged seed for *S. heptapotamica* was no germination at Ψ_S_ of −1.03 MPa. It is reported that the perianth-enclosed seed confined germination occurrence only after sufficient rainfall, which will dilute soil salinity and can also soften the perianth and leach the inhibitors (Rasheed et al., 2019).

## Conclusions

Seed germination of endemic species *S. heptapotamica, S. rosacea*, and *S. nitraria* were significantly inhibited by perianth and darkness and showed high sensitivity to different abiotic conditions. For better germination and seedling emergence, the perianth in these three species should be removed before sowing the seeds on the topsoil when the temperature is above 5/15°C. However, widespread species *S. ruthenica* seeds germinate equally well in light or dark in a wide range of temperatures. Considering the high tolerance of these *Salsola* species to salinity and drought, they could be cultivated for rehabilitating degraded arid-saline lands.

## Data Availability Statement

The raw data supporting the conclusions of this article will be made available by the authors, without undue reservation.

## Author Contributions

ZW and LW conceived the topic. PC and LJ performed the experiments and analyzed all statistical data. PC, LJ, WY, ZW, and LW wrote the manuscript. ZW and LW revised the manuscript. All authors contributed to the article and approved the submitted version.

## Funding

This work was supported by the Tianshan elite program of Xinjiang Uygur Autonomous Region (no. Y970000335), the National Natural Science Foundation of China (no. 31970354), and the Youth Innovation Promotion Association of Chinese Academy of Sciences (no. 2018479).

## Conflict of Interest

The authors declare that the research was conducted in the absence of any commercial or financial relationships that could be construed as a potential conflict of interest.

## Publisher's Note

All claims expressed in this article are solely those of the authors and do not necessarily represent those of their affiliated organizations, or those of the publisher, the editors and the reviewers. Any product that may be evaluated in this article, or claim that may be made by its manufacturer, is not guaranteed or endorsed by the publisher.

## References

[B1] Ahmed M. Z. Khan M. A. (2010). Tolerance and recovery responses of playa halophytes to light, salinity and temperature stresses during seed germination. Flora 205, 764–771. 10.1016/j.flora.2009.10.003

[B2] Al-Shamsi N. El-Keblawy A. Mosa K. A. Navarro T. (2018). Drought tolerance and germination response to light and temperature for seeds of saline and non-saline habitats of the habitat-indifferent desert halophyte *Suaeda vermiculata*. Acta Physiol. Plant. 40, 200. 10.1007/s11738-018-2771-z

[B3] Baskin C. C. Baskin J. M. (2014). Seeds: Ecology, Biogeography, and Evolution of Dormancy and Germination. 2nd ed. San Diego, CA: Academic Press.

[B4] Baskin J. M. Lu J. J. Baskin C. C. Tan D. Y. Wang L. (2014). Diaspore dispersal ability and degree of dormancy in heteromorphic species of cold deserts of northwest China: a review. Perspect. Plant Ecol. Evol. Syst. 16, 93–99. 10.1016/j.ppees.2014.02.004

[B5] Bhatt A. Perez-Garcia F. Caron M. M. Gallacher D. (2016). Germination response of *Salsola schweinfurthii* (Chenopodiaceae) to salinity and winged perianth removal. Seed Sci. Technol. 44, 1–7. 10.15258/sst.2016.44.2.14

[B6] Chang S. J. Zuo B. Wang X. W. Huang J. H. (2008). Influence of light, temperature and salt on the germination of *Salsola nitraria* Pall. Arid Land Geogr. 31, 897–903. 10.13826/j.cnki.cn65-1103/x.2008.06.002

[B7] Cresswell E. G. Grime J. P. (1981). Induction of a light requirement during seed development and its ecological consequences. Nature 291, 583–585. 10.1038/291583a0

[B8] D'Odorico P. Bhattachan A. Davis K. F. Ravi S. Runyan C. W. (2013). Global desertification: drivers and feedbacks. Adv. Water Resour. 51, 326–344. 10.1016/j.advwatres.2012.01.013

[B9] El-Keblawy A. (2017). Light and temperature requirements during germination of potential perennial grasses for rehabilitation of degraded sandy Arabian deserts. Land Degrad. Dev. 28, 1687–1695. 10.1002/ldr.2700

[B10] El-Keblawy A. Al-Ansari F. Al-Shamsi N. (2011). Effects of temperature and light on salinity tolerance during germination in two desert glycophytic grasses, *Lasiurus scindicus* and *Panicum turgidum*. Grass Forage Sci. 66, 173–182. 10.1111/j.1365-2494.2010.00773.x

[B11] El-Keblawy A. Bhatt A. Gairola S. (2013). Perianths color affects germination behaviour in wind-pollinated Salsola rubescens in Arabian deserts. Botany 92, 69–75. 10.1139/cjb-2013-0183

[B12] El-Keblawy A. Elnaggar A. Tammam A. Mosa K. A. (2020). Seed provenance affects salt tolerance and germination response of the habitat-indifferent *Salsola drummondii* halophyte in the arid Arabian deserts. Flora 266, 151592. 10.1016/j.flora.2020.151592

[B13] El-Keblawy A. Ksiksi T. (2005). Artificial forests as conservation sites for the native flora of the UAE. Forest Ecol. Manag. 213, 288–296. 10.1016/j.foreco.2005.03.058

[B14] Elnaggar A. El-Keblawy A. Mosa K. A. Navarro T. (2019). Adaptive drought tolerance during germination of *Salsola drummondii* seeds from saline and nonsaline habitats of the arid Arabian deserts. Botany 97, 122–123. 10.1139/cjb-2018-0174

[B15] Evans C. E. Etherington J. R. (1990). The effect of soil water potential on seed germination of some British plants. New Phytol. 115, 539–548. 10.1111/j.1469-8137.1990.tb00482.x 33874277

[B16] Guma I. R. Padron-Mederos M. A. Santos-Guerra A. Reyes-Betancort J. A. (2010). Effect of temperature and salinity on germination of *Salsola vermiculata* L. (Chenopodiaceae) from Canary Islands. J. Arid Environ. 74, 708–711. 10.1016/j.jaridenv.2009.10.001

[B17] Huang Z. Y. Zhang X. S. Zheng G. H. Gutterman Y. (2003). Influence of light, temperature, salinity and storage on seed germination of *Haloxylon ammodendron*. J. Arid Environ. 55, 453–464. 10.1016/S0140-1963(02)00294-X

[B18] Jurado E. Westoby M. Nelson D. (1991). Diaspore weight, dispersal, growth form and perenniality of central Australian plants. J. Ecol. 79, 811–828. 10.2307/2260669

[B19] Katerina K. Daws M. I. Thanos C. A. (2014). Campanulaceae: A family with small seeds that require light for germination. Ann. Bot-London 113, 135–143. 10.1093/aob/mct250 24232382 PMC3864721

[B20] Khan M. A. Gul B. (2006). “Halophyte seed germination,” in Ecophysiology of High Salinity Tolerant Plants eds M. A. Khan, and D. J. Weber (Dordrecht: Springer Press), 11–30. 10.1007/1-4020-4018-0_2

[B21] Khan M. A. Gul B. Weber D. J. (2002). Seed germination in the great basin halophyte *Salsola iberica*. Can. J. Bot. 80, 650–655. 10.1139/b02-046

[B22] Khan M. A. Gulzar S. (2003). Germination responses of *Sporobolus ioclados*: a saline desert grass. J. Arid Environ. 53, 387–394. 10.1006/jare.2002.1045

[B23] Khan M. A. Ungar I. A. (1984). The effect of salinity and temperature on the germination of polymorphic seeds and growth of *Atriplex triangularis* Willd. Am. J. Bot. 71, 481–489. 10.1002/j.1537-2197.1984.tb12533.x25855820

[B24] Kuang W. Yonggang M. A. Hong L. I. Liu C. (2014). Analysis of land degradation intensity and trend in Central Asia from 1999 to 2012. Remote Sens. Environ., 26, 163–169. 10.6046/gtzyyg.2014.04.26

[B25] Li Z. H. Luo P. (2003). SPSS for Windows: Statistical Analysis Tutorial. 2nd ed. Beijing: Electronic Industry Press, 257–265.

[B26] Lu Y. H. Ranjitkar S. Xu J. C. Ou X. K. Zhou Y. Z. (2016). Propagation of native tree species to restore subtropical evergreen broad-leaved forests in SW China. Forests 7, 12. 10.3390/f7010012

[B27] Ma Y. L. Wang J. Zhang J. H. Zhang S. Y. Liu Y. X. Lan H. Y. (2017). Seed heteromorphism and effects of light and abiotic stress on germination of a typical annual halophyte *Salsola ferganica* in cold desert. Front. Plant Sci. 8, 2257. 10.3389/fpls.2017.02257 29387073 PMC5776117

[B28] Maimaitijiang T. Ma Y. L. Lan H. Y. (2019). Effect of saltm coupled with drought stress on seed germination and seedling growth of *Salsola ferganica*. Arid Zone Res. 36, 878–885. 10.13866/j.azr.2019.04.11

[B29] Mamut J. Tan D. Y. Baskin C. C. Baskin J. M. (2019). Effects of water stress and NaCl stress on different life cycle stages of the cold desert annual *Lachnoloma lehmannii* in China. J. Arid Land 11, 774–784. 10.1007/s40333-019-0015-8

[B30] Michel B. E. Kaufmann M. R. (1973). The osmotic potential of polyethylene glycol 6000. Plant Physiol. 51, 914–916. 10.1104/pp.51.5.914 16658439 PMC366375

[B31] Naidoo G. Naicker K. (1992). Seed germination in the coastal halophytes *Triglochin bulbosa* and *Triglochin striata*. Aquat. Bot. 42, 217–229. 10.1016/0304-3770(92)90023-C

[B32] Negrão S. Schmöckel S. M. Tester M. (2017). Evaluating physiological responses of plants to salinity stress. Ann. Bot-London 119, 1–11. 10.1093/aob/mcw191 27707746 PMC5218372

[B33] Prince S. D. Becker-Reshef I. Rishmawi K. (2009). Detection and mapping of long-term land degradation using local net production scaling: application to Zimbabwe. Remote Sens. Environ. 113, 1046–1057. 10.1016/j.rse.2009.01.016

[B34] Probert R. J. (1992). “The role of temperature in germination ecophysiology,” in Seeds: the Ecology of Regeneration in plant Communities, ed M. Fenner (Wallingford: CABI Publishing), 410.

[B35] Qian J. Q. Liu Z. M. Hatier J. H. B. Liu B. (2016). The vertical distribution of soil seed bank and its restoration implication in an active sand dune of Northeastern inner Mongolia, China. Land Degrad. Dev. 27, 305–315. 10.1002/ldr.2428

[B36] Rasheed A. Hameed A. Gul B. Khan M. A. (2019). Perianth and abiotic factors regulate seed germination of *Haloxylon stocksii*—a cash crop candidate for degraded saline lands. Land Degrad. Dev. 30, 1468–1478. 10.1002/ldr.3334

[B37] Rasheed A. Hameed A. Khan M. A. Gul B. (2015). Effects of salinity, temperature, light and dormancy regulating chemicals on seed germination of *Salsola drummondii* Ulbr. Pak. J. Bot. 47, 11–19.

[B38] Ruan C. J. da Silva J. A. T. Mopper S. Qin P. Lutts S. (2010). Halophyte improvement for a salinized world. Crit. Rev. Plant Sci. 29, 329–359. 10.1080/07352689.2010.524517

[B39] Saatkamp A. Affre L. Baumberger T. Dumas P. J. Gasmi A. Gachet S.. (2011). Soil depth detection by seeds and diurnally fluctuating temperatures: different dynamics in 10 annual plants. Plant Soil 349, 331–340. 10.1007/s11104-011-0878-8

[B40] Sekmen A. H. Ozdemir F. Turkan I. (2004). Effects of salinity, light, and temperature on seed germination in a Turkish endangered halophyte, *Kalidiopsis wagenitzii* (Chenopodiaceae). Isr. J. Plant Sci. 52, 21–30. 10.1560/NXAR-71FB-CND5-E8FJ

[B41] Sen D. N. Chatterji U. N. (1968). Ecology of desert plants and observations on their seedlings. II. germination behaviour of seeds in Asclepiadaceae. Plant Syst. Evol. 115, 18–27. 10.1007/BF01373525

[B42] Song J. Fan H. Zhao Y. Jia Y. Du X. Wang B. (2008). Effect of salinity on germination, seedling emergence, seedling growth and ion accumulation of a euhalophyte Suaeda salsa in an intertidal zone and on saline inland. Aquat. Bot. 88, 331–337. 10.1016/j.aquabot.2007.11.004

[B43] Song J. Feng G. Tian C. Y. Zhang F. S. (2005). Strategies for adaptation of *Suaeda physophora, Haloxylon ammodendron* and *Haloxylon persicum* to a saline environment during seed-germination stage. Ann. Bot-London 96, 399–405. 10.1093/aob/mci196 16002418 PMC4246778

[B44] Tobe K. Li X. Omasa K. (2000). Effects of sodium chloride on seed germination and growth of two Chinese desert shrubs, *Haloxylon ammodendron* and H. persicum (Chenopodiaceae). Aust. J. Bot. 48, 455–460. 10.1071/BT99013

[B45] Wang C. Wang S. Fu B. J. Lu Y. H. Liu Y. X. Wu X. (2020). Integrating vegetation suitability in sustainable revegetation for the Loess Plateau, China. Sci. Total Environ. 11, 559–564. 10.1016/j.scitotenv.2020.143572 33213918

[B46] Wang Y. Jiang G. Q. Han Y. N. Liu M. M. (2013). Effects of salt, alkali and salt–alkali mixed stresses on seed germination of the halophyte *Salsola ferganica* (Chenopodiaceae). Acta Ecol. Sini. 33, 354–360. 10.1016/j.chnaes.2013.09.010

[B47] Wang Y. Zhang X. M. Li L. Li H. X. (2008). Seed on response of germination of four species of *Salsola* L. to main ecology factors. Seed 27, 58–63, 67. 10.16590/j.cnki.1001-4705.2008.08.061

[B48] Wei Y. Dong M. Huang Z. Y. (2007). Seed polymorphism, dormancy and germination of *Salsola affinis* (Chenopodiaceae), a dominant desert annual inhabiting the Junggar Basin of Xinjiang, China. Aust. J. Bot. 55, 464–470. 10.1071/BT06016

[B49] Wei Y. Dong M. Huang Z. Y. Tan D. Y. (2008). Factors influencing seed germination of *Salsola affinis* (Chenopodiaceae), a dominant annual halophyte inhabiting the deserts of Xinjiang, China. Flora 203, 134–140. 10.1016/j.flora.2007.02.003

[B50] Wei Y. Wang X. Y. (2006). Role of winged perianth in germination of *Haloxylon* (Chenopodiaceae) seeds. Acta Ecol. Sin. 26, 4014–4018.

[B51] Xing J. J. Cai M. Chen S. S. Chen L. Lan H. Y. (2013). Seed germination, plant growth and physiological responses of *Salsola ikonnikovii* to short-term NaCl stress. Plant Biosyst. 147, 285–297. 10.1080/11263504.2012.731017

[B52] Yu L. Guo T. D. Sun Z. C. Ma Y. P. Li Z. L. Zhao Y. N.. (2021). The seed germination characteristics and thresholds of two dominant plants in desert grassland-shrubland transition of the eastern Ningxia, China. Acta Ecol. Sin. 41, 4160–4169. 10.5846/stxb201910152147

[B53] Zhang K. Zheng H. Chen F. L. Ouyang Z. Y. Wang Y. Wu Y. F.. (2015). Changes in soil quality after converting *Pinus* to *Eucalyptus* plantations in southern China. Solid Earth 6, 115–123. 10.5194/se-6-115-2015

[B54] Zhu G. L. Mosyakin S. L. Clemants S. E. (2003). “Chenopodiaceae,” in Flora of China, eds Z. Y. Wu, and P. H. Raven (Beijing: Science Press), 351–414.

