# Supplementary material for: Seed Germination Response and Tolerance to Different Abiotic Stresses of Four Salsola Species Growing in an Arid Environment
Source: Front Plant Sci. 2022 May 19;13:892667. doi: 10.3389/fpls.2022.892667 (PMC9161727; doi:10.3389/fpls.2022.892667)
Supplement: Supplementary file 1 [file Data_Sheet_1.DOCX]

Supplementary Material

# Supplementary Tables

| **Supplementary TABLE 1.** Effects of winged perianths, light, temperature and their interactions on seed germination of the four *Salsola* plants*.* | | | | | | | | |
| --- | --- | --- | --- | --- | --- | --- | --- | --- |
| Parameter | *S. heptapotamica* | | *S. rosacea* | | *S. nitraria* | | *S. ruthenica* | |
|  | *Wald* | *P*-value | *Wald* | *P*-value | *Wald* | *P*-value | *Wald* | *P*-value |
| Winged perianth (Wp) | 54.679 | <0.001 | 47.267 | <0.001 | 66.978 | <0.001 | 13.365 | <0.001 |
| Light (L) | 36.975 | <0.001 | 55.134 | <0.001 | 22.019 | <0.001 | — | — |
| Temperature (T) | 42.165 | <0.001 | 28.362 | <0.001 | 53.442 | <0.001 | — | — |
| Wp × L | 4.356 | 0.037 | — | — | 9.920 | 0.002 | — | — |
| Wp × T | 15.533 | 0.004 | — | — | 114.943 | <0.001 | — | — |
| L × T | — | — | 22.664 | <0.001 | — | — | — | — |
| Wp × L × T | 25.300 | 0.004 | — | — | 49.419 | <0.001 | — | — |
| Note: — indicated that the parameters were eliminated in the model. | | | | | | | | |

| **Supplementary TABLE 2.** Effects of winged perianth, salinity and their interactions on seed germination and total germination of the four *Salsola* plants*.* | | | | | | | | | |
| --- | --- | --- | --- | --- | --- | --- | --- | --- | --- |
| Parameter | Factors | *S. heptapotamica* | | *S. rosacea* | | *S. nitraria* | | *S. ruthenica* | |
|  |  | *Wald* | *P*-value | *Wald* | *P*-value | *Wald* | *P*-value | *Wald* | *P*-value |
| Seed germination | Winged perianth (Wp) | — | — | — | — | — | — | 23.786 | < 0.001 |
|  | Salinity (S) | 196.045 | < 0.001 | 208.464 | < 0.001 | 129.116 | < 0.001 | 90.080 | < 0.001 |
|  | Wp × S | 16.331 | 0.006 | 23.076 | < 0.001 | 28.076 | < 0.001 | 78.080 | < 0.001 |
| Total germination | Winged perianth (Wp) | 31.615 | < 0.001 | 21.214 | < 0.001 | — | — | 14.473 | < 0.001 |
|  | Salinity (S) | 131.155 | < 0.001 | 135.367 | < 0.001 | 57.173 | < 0.001 | 67.603 | < 0.001 |
|  | Wp × S | 46.281 | < 0.001 | 35.349 | < 0.001 | 17.550 | 0.004 | 18.597 | 0.002 |
| Note: — indicated that the parameters were eliminated in the model. | | | | | | | | | |

| **Supplementary TABLE 3.** Effects of winged perianth, drought (PEG) and their interactions on seeds germination and total germination of the four *Salsola* plants*.* | | | | | | | | | |
| --- | --- | --- | --- | --- | --- | --- | --- | --- | --- |
| Parameter | Factors | *S. heptapotamica* | | *S. rosacea* | | *S. nitraria* | | *S. ruthenica* | |
|  |  | *Wald* | *P*-value | *Wald* | *P*-value | *Wald* | *P*-value | *Wald* | *P*-value |
| Seed germination | Winged perianth (Wp) | 126.711 | < 0.001 | 11.630 | 0.001 | 54.667 | < 0.001 | 10.281 | 0.001 |
|  | PEG (P) | 287.383 | < 0.001 | 203.285 | < 0.001 | 240.237 | < 0.001 | 324.091 | < 0.001 |
|  | Wp × P | — | — | — | — | — | — | — | — |
| Total germination | Winged perianth (Wp) | 38.623 | < 0.001 | — | — | 22.637 | < 0.001 | 9.825 | 0.002 |
|  | PEG (P) | 44.634 | < 0.001 | 222.023 | < 0.001 | — | — | 14.070 | 0.015 |
|  | Wp × P | — | — | — | — | — | — | — | — |
| Note: — indicated that the parameters were eliminated in the model. | | | | | | | | | |

# Supplementary Figures

**
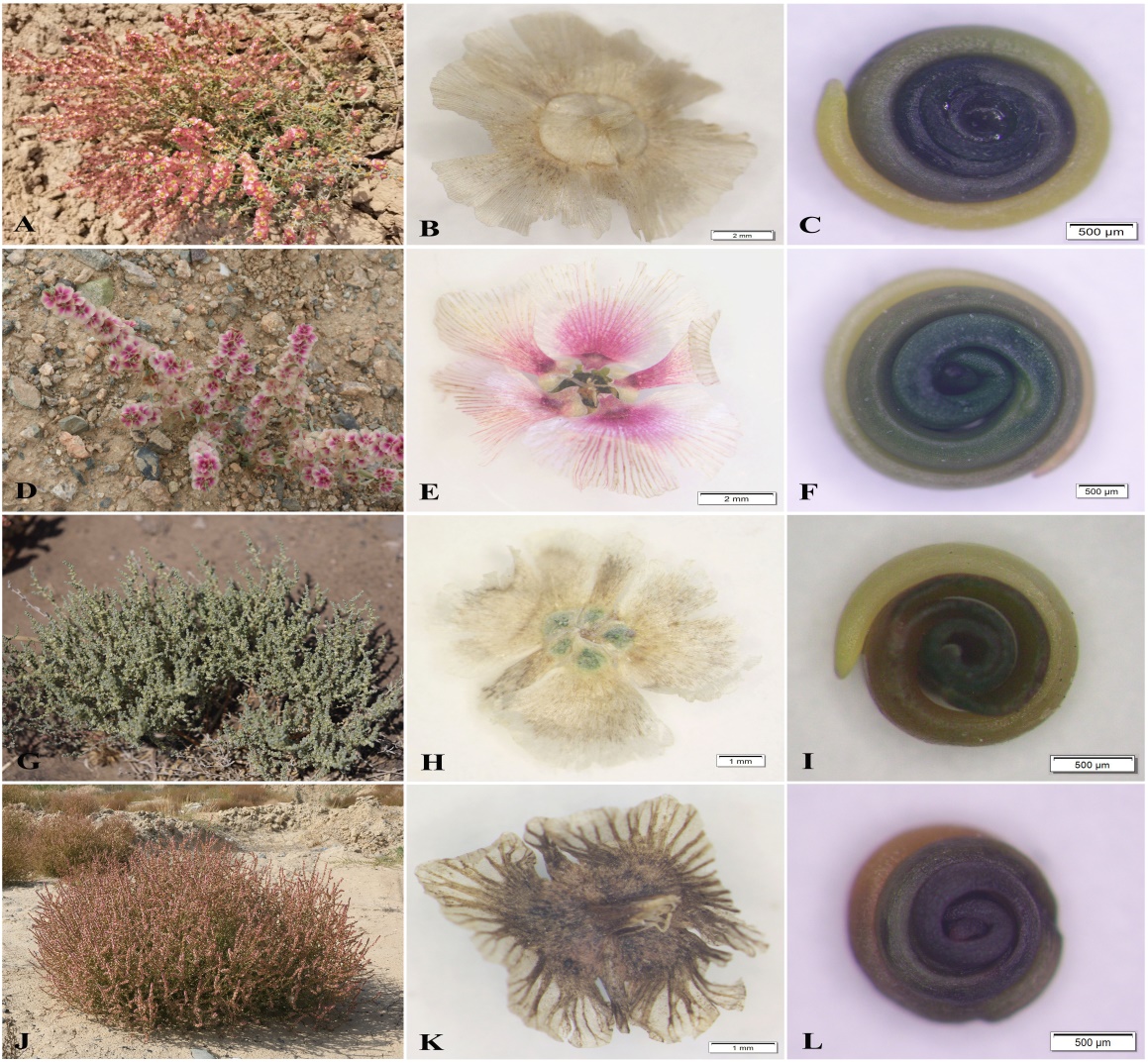
**

**Supplementary FIGURE 1.** Habitats of the four *Salsola* plants, and morphologies of their winged seeds and naked seeds. (A-C) *S. heptapotamica*, (D-F) *S. rosacea*, (G-I) *S. nitraria*, (J-L) *S. ruthenica*.


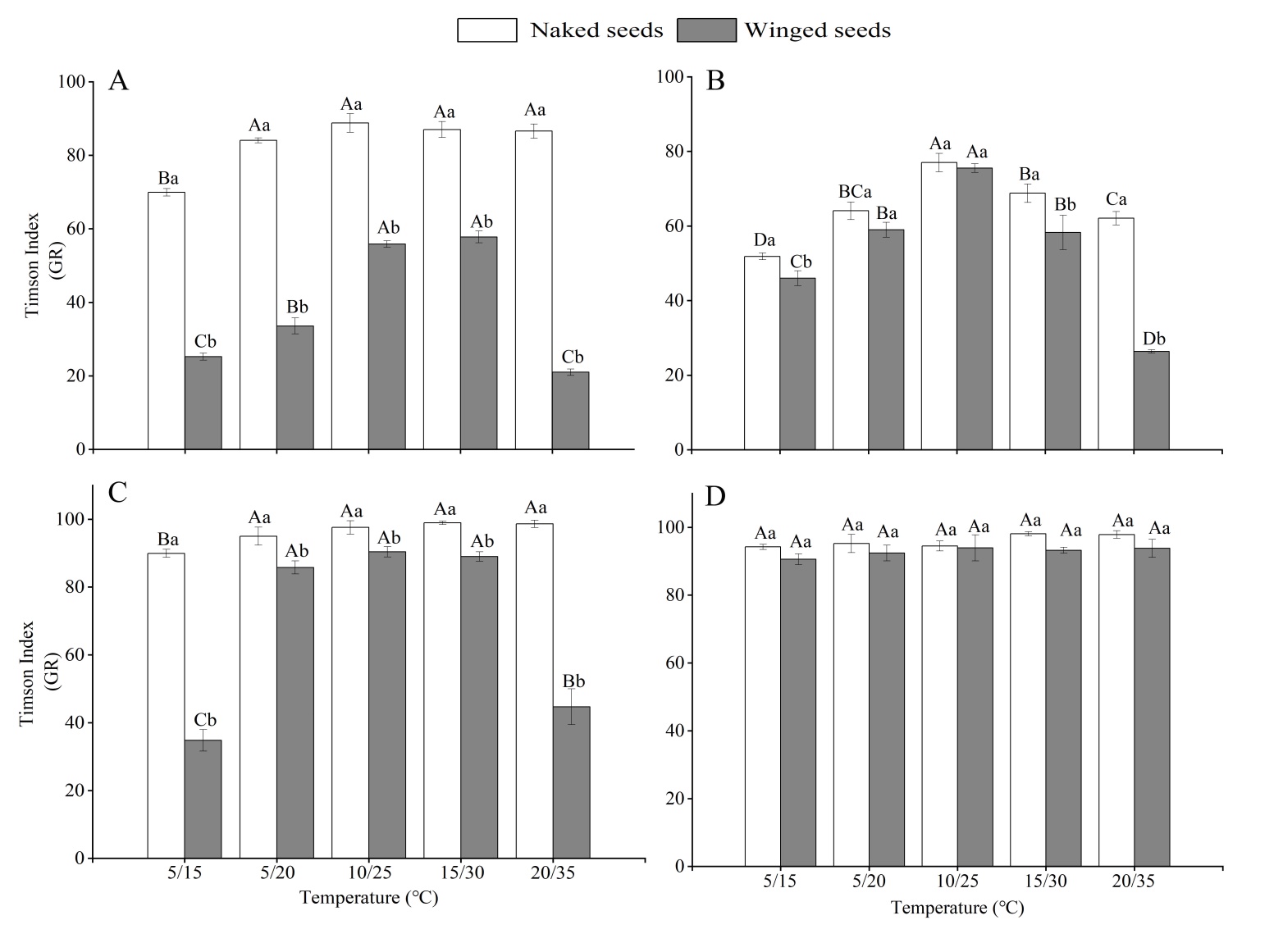


**Supplementary FIGURE 2.** The effects of winged perianth and temperature on seed germination index (mean ± SE) of these four *Salsola* plants. (A) *S. heptapotamica*, (B) *S. rosacea*, (C) *S. nitraria*, (D) *S. ruthenica*. Different uppercase letters denote significant difference (*P* < 0.05) of germination percentage at different temperature for the same perianth treatment, and different lowercase letters indicate significant difference (*P* < 0.05) of germination percentage for different treatments of perianth at the same temperature.
